# Correlation between frailty status and prolonged length of stay in the post-anesthesia care unit and development of a predictive model in elderly patients undergoing painless gastrointestinal endoscopy

**DOI:** 10.3389/fmed.2026.1905761

**Published:** 2026-08-10

**Authors:** Zhilin Chen, Jinguang Zhang, Wudong Zhuang, Fang Xing, Jianxi Zhou, Dongxu Sun

**Affiliations:** 1 Department of Endoscopy, Cangzhou Hospital of Integrated Traditional Chinese and Western Medicine, Cangzhou, Hebei, China; 2 Department of Anesthesiology, Cangzhou Hospital of Integrated Traditional Chinese and Western Medicine, Cangzhou, Hebei, China

**Keywords:** frailty, length of stay, painless gastrointestinal endoscopy, post-anesthesia care unit, predictive model

## Abstract

**Background:**

The incidence of prolonged length of stay in the post-anesthesia care unit (PACU) is high among elderly patients undergoing painless gastrointestinal endoscopy, and the value of frailty assessment for perioperative risk stratification in this population remains unclear.

**Methods:**

A total of 500 elderly patients undergoing outpatient painless gastrointestinal endoscopy were enrolled. Preoperative frailty was assessed using the Fried Frailty Phenotype, and perioperative clinical data were collected. Independent risk factors for prolonged PACU length of stay were identified through Logistic regression analysis, and a predictive model was constructed.

**Results:**

Frailty severity showed a dose-dependent relationship with the incidence of intraoperative and postoperative adverse events and PACU length of stay. Multivariable analysis revealed that frailty was the strongest independent risk factor for prolonged PACU length of stay (OR = 4.278, 95%CI: 2.207-8.286, *P* < 0.001). The constructed multivariable predictive model had an area under the curve of 0.804, with a sensitivity of 82.4% and a specificity of 77.1%.

**Conclusions:**

Frailty significantly increases the risk of prolonged PACU length of stay in elderly patients undergoing painless gastrointestinal endoscopy. The predictive model based on frailty status has favorable clinical application value.

## 1 Introduction

With the acceleration of global population aging, the proportion of the population aged 65 years and older continues to rise, and China has entered a deeply aging society. The demand for endoscopic examination and treatment among elderly patients is growing exponentially (1, 2). Painless gastrointestinal endoscopy eliminates intraoperative discomfort through intravenous anesthesia, significantly improving patient compliance and the quality of endoscopic procedures, and has become the preferred method for screening and diagnosis of gastrointestinal diseases in the elderly population (3, 4). However, elderly patients are characterized by multisystem physiological degenerative changes, multiple chronic comorbidities, and impaired drug metabolism, resulting in significantly higher risks associated with anesthesia and endoscopic procedures compared with younger individuals (5, 6).

The post-anesthesia care unit (PACU) is a critical link in ensuring patient safety after surgery (7). Prolonged PACU length of stay not only increases medical resource consumption and patient economic burden but is also associated with elevated risks of postoperative adverse events such as pulmonary infection, deep vein thrombosis, and cognitive dysfunction (8–10). Previous studies have shown that the incidence of prolonged PACU stay after painless gastrointestinal endoscopy in elderly patients can reach 20%−40%, which has become a common clinical concern for both anesthesiologists and endoscopy centers (11). Identifying high-risk factors for prolonged PACU stay and implementing targeted interventions are of great significance for optimizing perioperative management and improving the efficiency of medical resource utilization.

Traditional preoperative risk assessment tools, such as the American Society of Anesthesiologists (ASA) classification, are primarily based on subjective judgments of patients' comorbidities and general status, making it difficult to accurately identify occult declines in physiological reserve in elderly patients (12, 13). Frailty is a geriatric syndrome characterized by reduced multisystem physiological reserve and increased susceptibility to stress events, and has been confirmed as an independent risk factor for predicting perioperative adverse outcomes in surgical patients (14, 15). In recent years, the application of frailty assessment in the field of anesthesiology has received increasing attention, and multiple studies have demonstrated that frailty increases the risk of adverse events after general anesthesia in elderly patients (16, 17).

In the field of digestive endoscopy, preliminary studies have confirmed that frailty is associated with intraoperative adverse events such as hypotension and hypoxemia in elderly patients undergoing painless gastrointestinal endoscopy (18, 19). However, most existing studies on frailty and perioperative outcomes in this population have focused on intraoperative adverse events, with relatively insufficient attention to the postoperative PACU recovery process. Meanwhile, the majority of studies have analyzed frailty as a dichotomous variable, lacking systematic comparisons between pre-frailty and different frailty strata, which makes it difficult to fully reveal the dose-response relationship between frailty severity and perioperative outcomes (20). Furthermore, most existing studies are single-center with small sample sizes, and no study has yet constructed a predictive model for prolonged PACU length of stay based on frailty status, which cannot meet the clinical needs for individualized risk stratification and intervention. Differences in the definition of prolonged PACU stay across studies have also limited the comparability of research results (21). Clarifying the characteristics of PACU recovery in elderly patients with different frailty statuses and identifying independent risk factors for prolonged PACU stay have important clinical value for developing individualized anesthesia regimens and optimizing postoperative resuscitation processes.

This study enrolled 500 elderly patients undergoing painless gastrointestinal endoscopy, applied the Fried Frailty Phenotype assessment criteria and a unified definition of prolonged PACU stay, and systematically analyzed differences in baseline characteristics, intraoperative anesthesia and procedure parameters, PACU recovery outcomes, and adverse event incidence among patients with different frailty statuses. Univariate and multivariable logistic regression analyses were performed to identify independent risk factors for prolonged PACU length of stay, and a predictive model was constructed to evaluate its clinical application value.

## 2 Materials and methods

### 2.1 Study design and population

This was a single-center retrospective cohort study. All clinical data included in this study were prospectively and standardized collected during routine clinical practice and documented in the hospital electronic medical record system. We retrospectively extracted and analyzed data from 500 eligible elderly patients undergoing outpatient painless gastrointestinal endoscopy at the Endoscopy Center of Cangzhou Hospital of Integrated Traditional Chinese and Western Medicine, Hebei Province, between January 2025 and December 2025. No intervention to the original clinical workflow was implemented during the study period. This study was approved by the Ethics Committee of Cangzhou Hospital of Integrated Traditional Chinese and Western Medicine. Given the retrospective observational design, the use of de-identified routine clinical data, and no additional intervention or risk to patients, the ethics committee waived the requirement for specific written informed consent for study participation.

Inclusion criteria: (1) age ≥ 65 years. (2) Undergoing simple painless gastroscopy, simple painless colonoscopy, or combined painless gastroscopy and colonoscopy. (3) American Society of Anesthesiologists (ASA) physical status classification I-III. (4) Ability to communicate normally and cooperate with preoperative frailty assessment. (5) Signed written informed consent for painless gastrointestinal endoscopy and intravenous anesthesia in accordance with routine clinical requirements.

Exclusion criteria: (1) undergoing invasive therapeutic procedures such as endoscopic polypectomy, esophageal foreign body removal, hemostasis, endoscopic mucosal resection (EMR), or endoscopic submucosal dissection (ESD); (2) inpatients (rationale: inpatients are directly transferred to the ward after surgery and do not enter the routine outpatient PACU resuscitation process. Their baseline status, anesthesia management, and postoperative management are essentially different from those of outpatients, and exclusion significantly improves study homogeneity); (3) presence of language communication disorders, intellectual disability, or history of mental illness; (4) severe musculoskeletal disorders or limb disability that prevent completion of grip strength and walking speed tests; (5) use of sedatives, analgesics, or antihistamines within 24 h before surgery; (6) allergy to anesthetic drugs used in this study, including propofol, remifentanil, and midazolam; (7) presence of uncontrolled severe arrhythmia, acute heart failure, or respiratory failure before surgery.

### 2.2 Data collection and quality control

A research team consisting of three anesthesiologists and two gastroenterologists who had received unified training and passed the assessment extracted all data from the hospital electronic medical record system and pre-established dedicated case report forms (CRFs) during the study period. All data were entered into an Excel database using the double data entry method by two independent researchers, cross-checked for accuracy, and then locked. Variables with missing values >5% were excluded. After calculation, all core analysis variables had a missing rate ≤ 5%, so no variables were excluded due to excessive missingness, and no patients were removed from the final cohort for missing data. Detailed missing rates of each variable before imputation are presented in Supplementary Table S2. For continuous variables with missing values ≤ 5%, mean imputation was performed, and for categorical variables, mode imputation was used.

Data collected included: Baseline data: age, sex, BMI, ASA classification, smoking history (defined as cumulative smoking ≥100 cigarettes), drinking history (defined as weekly drinking ≥1 time for ≥1 year), hypertension, type 2 diabetes mellitus, coronary heart disease, chronic obstructive pulmonary disease, history of cerebrovascular disease, and number of comorbidities; Intraoperative data: total and per-kilogram doses of propofol, remifentanil, and midazolam; gastroscopy procedure duration (time from endoscope insertion to withdrawal from the digestive tract), colonoscopy procedure duration, total procedure duration (time from anesthesia induction to patient awakening); incidence of various intraoperative adverse events and corresponding management measures; Postoperative PACU data: Aldrete score immediately upon PACU admission, PACU length of stay (time from PACU admission to meeting discharge criteria), time to achieve Aldrete score of 9, Aldrete score at PACU discharge, incidence of various adverse events in the PACU, number of adverse events requiring pharmacological intervention, and discharge disposition.

### 2.3 Preoperative frailty assessment

Preoperative frailty assessment was a routine preoperative evaluation item for elderly patients scheduled for painless endoscopy in our center, and had been incorporated into the standardized clinical management protocol. The assessment was performed 1 day before surgery using the internationally recognized Fried Frailty Phenotype criteria. All assessments were performed by the same anesthesiologist who had received standardized training, in a quiet and well-lit environment. The assessment procedure was explained in detail to the patients before the evaluation. The assessment content and diagnostic criteria were as follows: (1) unintentional weight loss: ≥5% body weight loss in the past 1 year (verified by weight records in outpatient medical records); (2) fatigue: patient self-report of “feeling low energy most or all of the time in the past month” using relevant items from the FRAIL scale; (3) decreased grip strength: maximum grip strength of the dominant hand was measured using a calibrated electronic dynamometer, with three consecutive measurements taken and the maximum value recorded. Cutoff values were < 27 kg for men and < 18 kg for women; (4) slowed walking speed: time to walk 4 m on flat ground was measured, with a walking speed < 0.8 m/s (patients wore their usual shoes and did not use assistive devices); (5) decreased physical activity: assessed using the short form of the International Physical Activity Questionnaire (IPAQ). Physical activity levels below 383 kcal/week for men and 270 kcal/week in the past week were considered decreased. Patients were divided into three groups based on assessment results: non-frail group (0 abnormal indicators), pre-frail group (1–2 abnormal indicators), and frail group (≥3 abnormal indicators).

### 2.4 Anesthesia and endoscopic procedure protocols

All procedures were performed in strict accordance with the Chinese Guidelines for Sedation and Anesthesia in Digestive Endoscopy (22) and the Chinese Guidelines for Perioperative Anesthesia Management of Elderly Patients (23).

Preoperative preparation: patients fasted from solid food for 8 h and abstained from clear liquids for 2 h before surgery. After entering the operating room, an upper extremity peripheral venous access was established, and non-invasive blood pressure (NIBP), heart rate (HR), electrocardiogram (ECG), and pulse oxygen saturation (SpO_2_) were routinely monitored, with baseline vital signs recorded.

Anesthesia administration: anesthesia induction: remifentanil was first administered via continuous infusion at 0.05–0.1 μg·kg^−1^·min^−1^, followed by slow intravenous injection of propofol 1.5–2.5 mg/kg (injection speed 20–40 mg/10 s). Endoscopic procedures were initiated after the patient's eyelash reflex disappeared and they became unresponsive to verbal commands. Anesthesia maintenance: the remifentanil infusion rate was adjusted to 0.05–0.2 μg·kg^−1^·min^−1^ based on patient movement responses, vital sign changes, and bispectral index (BIS, maintained at 40–60). Propofol 20–30 mg was administered intermittently as needed. A uniform titration protocol was applied to all patients, with no pre-specified dose reduction for patients with frailty. For patients with anxiety or prolonged procedure duration, midazolam 1–2 mg was administered intravenously for adjunctive sedation if necessary.

Endoscopic procedures: all endoscopic procedures were performed by gastroenterologists with more than 5 years of clinical experience. The patient's airway was kept patent throughout the procedure, and oxygen was administered continuously via face mask at a flow rate of 2–3 L/min.

Definition and management of intraoperative adverse events: (1) hypotension: systolic blood pressure < 90 mmHg or >30% decrease from baseline, treated with intravenous ephedrine 6–12 mg; (2) bradycardia: heart rate < 50 beats per min, treated with intravenous atropine 0.3–0.5 mg; (3) movement reaction: limb movement affecting endoscopic procedures, treated with additional propofol 20–30 mg; (4) cough: severe cough affecting ventilation, treated with temporary cessation of the procedure and face mask oxygen supplementation; (5) hypoxemia: SpO_2_ < 90% lasting >10 s, treated with face mask oxygen supplementation, and endotracheal intubation for assisted ventilation if necessary.

### 2.5 PACU management and outcome measures

PACU management protocol: patients were transferred to the PACU immediately after surgery and monitored by dedicated PACU nurses with more than 3 years of work experience. NIBP, HR, ECG, and SpO_2_ were continuously monitored. The Aldrete Post-Anesthesia Recovery Score was used to assess the patient's recovery status (24), which was evaluated immediately upon PACU admission, every 15 min thereafter, and before PACU discharge. PACU discharge criteria: Aldrete score ≥ 9, stable vital signs (systolic blood pressure 90–160 mmHg, heart rate 50–100 beats per minute, SpO_2_ ≥ 95%) maintained for more than 15 min, and no significant nausea, vomiting, dizziness, or other discomfort.

Outcome measures: primary outcome measure: prolonged PACU length of stay, defined as PACU length of stay ≥35 min (the 75th percentile of PACU length of stay in the total study population). This percentile-based definition was selected to minimize subjectivity in threshold setting; the 35-min cutoff corresponds to the upper limit of routine PACU recovery duration in our center, and is associated with increased postoperative adverse events and reduced bed turnover efficiency. Secondary outcome measures: incidence of various intraoperative adverse events, incidence of various adverse events in the PACU, Aldrete score immediately upon PACU admission, time to achieve Aldrete score of 9, incidence of adverse events requiring pharmacological intervention, and discharge disposition (direct home/prolonged observation/transfer to inpatient ward).

### 2.6 Statistical analysis

All statistical analyses were performed using SPSS 26.0 (IBM Corp., Armonk, NY, United States) statistical software. Normally distributed continuous variables were expressed as mean ± standard deviation (*x* ± *s*), with intergroup comparisons using one-way analysis of variance (ANOVA) and pairwise comparisons using the LSD-*t* test. Non-normally distributed continuous variables were expressed as median (P25, P75), with intergroup comparisons using the Kruskal–Wallis *H* test and pairwise comparisons using Bonferroni correction. Categorical variables were expressed as number (percentage) [*n* (%)], with intergroup comparisons using the chi-square test and pairwise comparisons using Bonferroni correction (test level α' = 0.0167).

With prolonged PACU length of stay as the dependent variable, variables with *P* < 0.05 in univariate analysis were initially screened. Collinearity diagnosis was performed using the variance inflation factor (VIF), with a VIF > 5 defined as significant collinearity. Eligible variables were then included in a multivariable stepwise Logistic regression model (forward method, inclusion criterion *P* < 0.05, exclusion criterion *P* > 0.10) to identify independent risk factors, which could further minimize the impact of collinearity during variable selection. The discriminative performance of the multivariable predictive model was evaluated using the receiver operating characteristic (ROC) curve, with calculation of the area under the curve (AUC) and 95% confidence interval (CI). The optimal cutoff value and corresponding sensitivity and specificity were determined using the Youden index (sensitivity + specificity – 1). No internal or external validation of the predictive model was performed in this study; the reported performance reflects the apparent discriminative ability in the derivation cohort. All tests were two-sided, and a *P*-value < 0.05 was considered statistically significant.

## 3 Results

### 3.1 Baseline clinical characteristics of patients with different frailty statuses

There were statistically significant intergroup differences in age, body mass index (BMI), American Society of Anesthesiologists (ASA) classification, prevalence of various chronic diseases, and distribution of the number of comorbidities among the three groups (all *P* < 0.001). No statistically significant intergroup differences were observed in sex distribution, smoking and drinking history, or type of endoscopic examination (*P* = 0.760, 0.404, 0.094, and 0.985, respectively). Patient age increased progressively with higher frailty grades, while BMI showed a synchronous decreasing trend. The proportion of ASA class I patients gradually decreased from the non-frail to the frail group, while the proportion of ASA class III patients gradually increased. The prevalence of hypertension, type 2 diabetes mellitus, coronary heart disease, chronic obstructive pulmonary disease, and cerebrovascular disease continued to rise with frailty progression. The median number of comorbidities was 1(0,1), 1(0,2), and 2(1,3) in the non-frail, pre-frail, and frail groups, respectively. The proportion of patients with no comorbidities gradually decreased, while the proportion of patients with ≥3 comorbidities gradually increased. The composition ratios of the three endoscopic examination methods were similar among all groups (Table 1).

**Table 1:** Baseline characteristics of elderly patients undergoing painless gastrointestinal endoscopy by frailty status.

Characteristic	Total population (*n* = 500)	Non-frail group (*n* = 202)	Pre-frail group (*n* = 198)	Frail group (*n* = 100)	*P* value
Demographic characteristics					
Age (years), mean ± SD	72.9 ± 5.5	70.3 ± 4.2	73.4 ± 5.1	76.8 ± 4.7	< 0.001
Sex, *n* (%)					0.760
Male	261 (52.2)	106 (52.5)	100 (50.5)	55 (55.0)	
Female	239 (47.8)	96 (47.5)	98 (49.5)	45 (45.0)	
BMI (kg/m^2^), mean ± SD	24.1 ± 2.3	24.7 ± 2.1	24.0 ± 2.2	23.2 ± 2.4	< 0.001
ASA classification, *n* (%)					< 0.001
Class I	126 (25.2)	72 (35.6)	46 (23.2)	8 (8.0)	
Class II	312 (62.4)	122 (60.4)	128 (64.6)	62 (62.0)	
Class III	62 (12.4)	8 (4.0)	24 (12.1)	30 (30.0)	
Lifestyle habits, *n* (%)					
Smoking history					0.404
Yes	185 (37.0)	68 (33.7)	76 (38.4)	41 (41.0)	
No	315 (63.0)	134 (66.3)	122 (61.6)	59 (59.0)	
Drinking history					0.094
Yes	161 (32.2)	54 (26.7)	70 (35.4)	37 (37.0)	
No	339 (67.8)	148 (73.3)	128 (64.6)	63 (63.0)	
Comorbidities, *n* (%)					
Hypertension					< 0.001
Yes	286 (57.2)	98 (48.5)	114 (57.6)	74 (74.0)	
No	214 (42.8)	104 (51.5)	84 (42.4)	26 (26.0)	
Type 2 diabetes mellitus					0.001
Yes	124 (24.8)	36 (17.8)	50 (25.3)	38 (38.0)	
No	376 (75.2)	166 (82.2)	148 (74.7)	62 (62.0)	
Coronary heart disease					< 0.001
Yes	86 (17.2)	22 (10.9)	34 (17.2)	30 (30.0)	
No	414 (82.8)	180 (89.1)	164 (82.8)	70 (70.0)	
Chronic obstructive pulmonary disease					< 0.001
Yes	68 (13.6)	16 (7.9)	24 (12.1)	28 (28.0)	
No	432 (86.4)	186 (92.1)	174 (87.9)	72 (72.0)	
History of cerebrovascular disease					< 0.001
Yes	42 (8.4)	6 (3.0)	16 (8.1)	20 (20.0)	
No	458 (91.6)	196 (97.0)	182 (91.9)	80 (80.0)	
Number of comorbidities, *n* (%)					< 0.001
0	122 (24.4)	78 (38.6)	40 (20.2)	4 (4.0)	
1	185 (37.0)	76 (37.6)	81 (40.9)	28 (28.0)	
2	123 (24.6)	36 (17.8)	53 (26.8)	34 (34.0)	
≥3	70 (14.0)	12 (5.9)	24 (12.1)	34 (34.0)	
Number of comorbidities, median (P25, P75)	1 (0, 2)	1 (0, 1)	1 (0, 2)	2 (1, 3)	< 0.001
Type of examination, *n* (%)					0.985
Isolated painless gastroscopy	152 (30.4)	62 (30.7)	60 (30.3)	30 (30.0)	
Isolated painless colonoscopy	198 (39.6)	82 (40.6)	76 (38.4)	40 (40.0)	
Combined painless gastroscopy and colonoscopy	150 (30.0)	58 (28.7)	62 (31.3)	30 (30.0)	

BMI, body mass index; ASA, American Society of Anesthesiologists. Continuous variables are presented as mean ± SD or median (interquartile range), and categorical variables as *n* (%). For normally distributed continuous variables, one-way ANOVA was used for intergroup comparisons; for non-normally distributed continuous variables, Kruskal–Wallis *H* test was used; for categorical variables, chi-square test was used.

### 3.2 Intraoperative anesthetic drug dosage, procedure duration, and intraoperative adverse events

**Figure 1:**
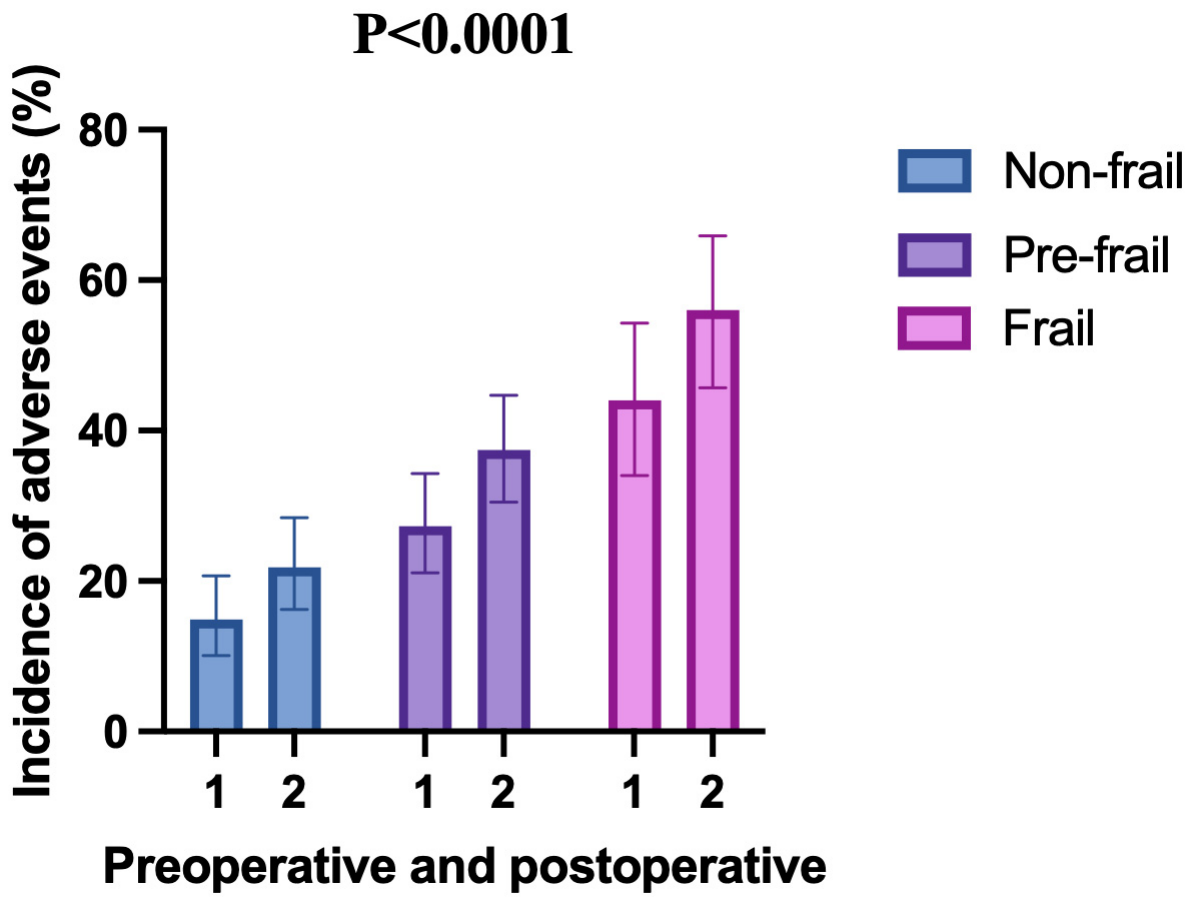
Incidence of total intraoperative and postoperative adverse events by frailty stratum.

There were statistically significant intergroup differences in the total and per-kilogram doses of propofol and remifentanil (all *P* < 0.001), with dosages of these drugs decreasing with increasing frailty severity. No statistically significant intergroup differences were observed in midazolam usage rate or single-dose administration (*P* = 0.184 and 0.428, respectively). Gastroscopy, colonoscopy, and total procedure durations were prolonged with higher frailty grades, with intergroup *P* = 0.017 for gastroscopy duration and *P* < 0.001 for both colonoscopy and total procedure durations. The incidence of intraoperative hypotension, bradycardia, movement, cough, and hypoxemia gradually increased with frailty severity, with intergroup comparisons showing *P* < 0.001, *P* = 0.001, *P* < 0.001, *P* = 0.001, and *P* = 0.005, respectively. Stratified by the frequency of intraoperative adverse events, the proportion of patients with no adverse events decreased from 85.1% in the non-frail group to 56.0% in the frail group, while the proportion of patients with ≥2 intraoperative adverse events increased from 1.0% to 16.0%, with an intergroup *P* < 0.001 (Table 2). The overall incidence of total intraoperative adverse events was statistically significantly different among the three groups (χ^2^ = 25.84, df = 2, *P* < 0.001). Pairwise comparisons after Bonferroni correction all met the preset test level. The incidence of total intraoperative adverse events was 14.9% (95% CI: 10.1%−20.7%), 27.3% (95% CI: 21.1%−34.3%), and 44.0% (95% CI: 34.0%−54.3%) in the non-frail, pre-frail, and frail groups, respectively (Figure 1).

**Table 2:** Intraoperative anesthesia and procedure characteristics of patients by frailty status.

Characteristic	Total population (*n* = 500)	Non-frail group (*n* = 202)	Pre-frail group (*n* = 198)	Frail group (*n* = 100)	*P* value
Anesthetic drug dosages					
Total propofol dose (mg), mean ± SD	142.3 ± 29.7	156.8 ± 26.3	139.5 ± 28.1	121.7 ± 27.4	< 0.001
Propofol dose per kg body weight (mg/kg), mean ± SD	2.2 ± 0.4	2.4 ± 0.3	2.2 ± 0.4	1.9 ± 0.3	< 0.001
Total remifentanil dose (μg), mean ± SD	42.6 ± 9.8	46.3 ± 8.9	41.8 ± 9.5	37.2 ± 10.1	< 0.001
Remifentanil dose per kg body weight (μg/kg), mean ± SD	0.67 ± 0.15	0.73 ± 0.13	0.66 ± 0.14	0.58 ± 0.16	< 0.001
Midazolam usage rate, *n* (%)					0.184
Yes	56 (11.2)	18 (8.9)	22 (11.1)	16 (16.0)	
No	444 (88.8)	184 (91.1)	176 (88.9)	84 (84.0)	
Total midazolam dose (mg), mean ± SD	1.6 ± 0.5	1.7 ± 0.4	1.6 ± 0.5	1.5 ± 0.5	0.428
Procedure duration					
Gastroscopy procedure duration (min), mean ± SD	6.7 ± 2.0	6.4 ± 1.8	6.8 ± 2.0	7.1 ± 2.2	0.017
Colonoscopy procedure duration (min), mean ± SD	12.2 ± 4.0	11.5 ± 3.6	12.3 ± 3.9	13.5 ± 4.3	< 0.001
Total procedure duration (min), mean ± SD	14.9 ± 4.5	13.9 ± 4.0	15.0 ± 4.4	16.5 ± 4.8	< 0.001
Intraoperative adverse events, *n* (%)					
Hypotension (SBP<90 mmHg or >30% decrease from baseline)					< 0.001
Yes	74 (14.8)	17 (8.4)	26 (13.1)	31 (31.0)	
No	426 (85.2)	185 (91.6)	172 (86.9)	69 (69.0)	
Bradycardia (HR<50 beats/min)					0.001
Yes	30 (6.0)	6 (3.0)	10 (5.1)	14 (14.0)	
No	470 (94.0)	196 (97.0)	188 (94.9)	86 (86.0)	
Movement reaction (affecting procedure)					< 0.001
Yes	42 (8.4)	10 (5.0)	14 (7.1)	18 (18.0)	
No	458 (91.6)	192 (95.0)	184 (92.9)	82 (82.0)	
Cough					0.001
Yes	36 (7.2)	8 (4.0)	12 (6.1)	16 (16.0)	
No	464 (92.8)	194 (96.0)	186 (93.9)	84 (84.0)	
Hypoxemia (SpO_2_<90% lasting > 10 s)					0.005
Yes	16 (3.2)	2 (1.0)	6 (3.0)	8 (8.0)	
No	484 (96.8)	200 (99.0)	192 (97.0)	92 (92.0)	
Total intraoperative adverse events, *n* (%)					< 0.001
0 events	372 (74.4)	172 (85.1)	144 (72.7)	56 (56.0)	
1 event	102 (20.4)	28 (13.9)	46 (23.2)	28 (28.0)	
≥2 events	26 (5.2)	2 (1.0)	8 (4.0)	16 (16.0)	

SBP, systolic blood pressure; HR, heart rate; SpO_2_, pulse oxygen saturation. Continuous variables are presented as mean ± SD, and categorical variables as *n* (%). For normally distributed continuous variables, one-way ANOVA was used for intergroup comparisons; for categorical variables, chi-square test was used.

### 3.3 PACU recovery indicators, postoperative adverse events, and discharge disposition

**Figure 2:**
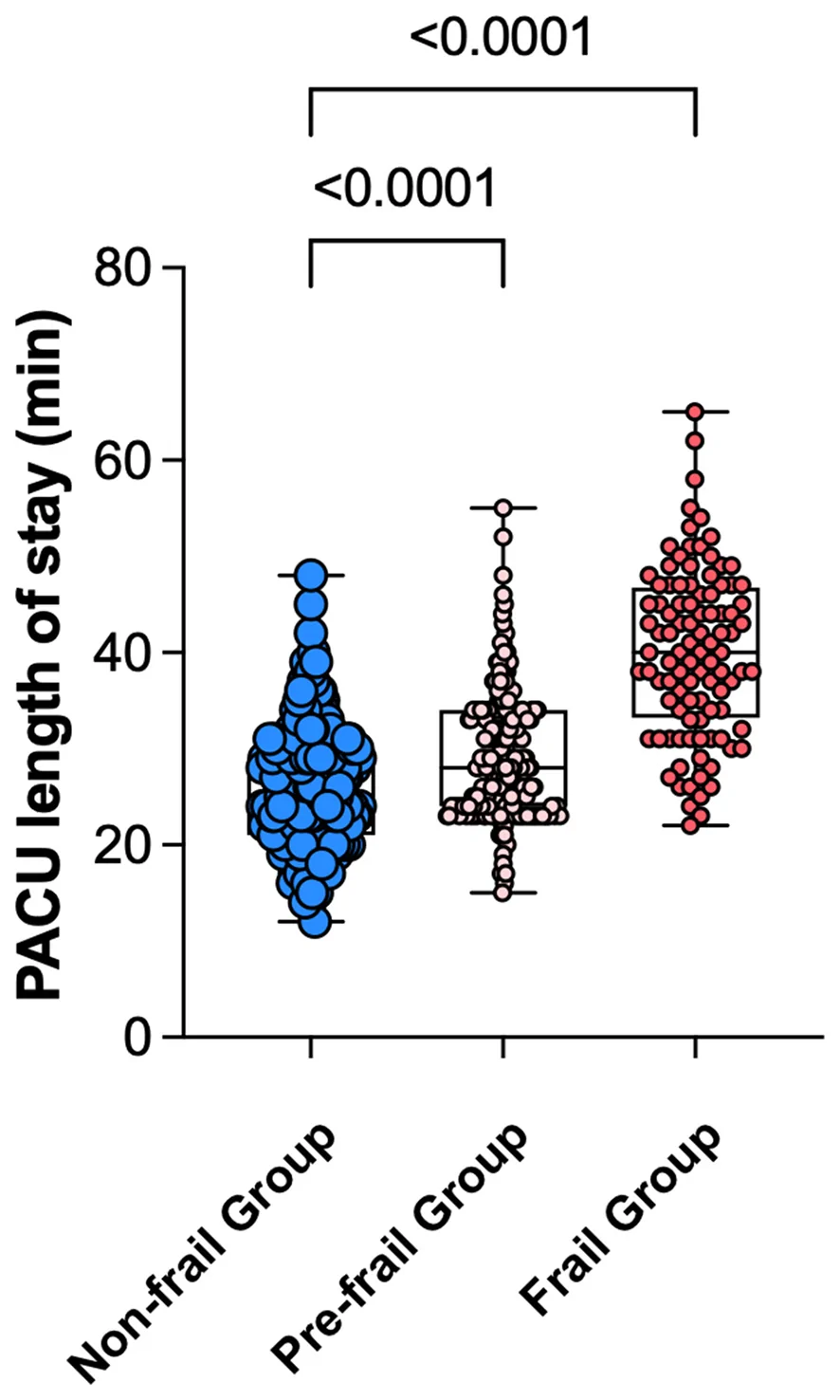
Distribution of PACU length of stay by frailty stratum.

The median PACU length of stay was 24(20,29), 28(23,34), and 38(31,47) min in the three groups, respectively, with an overall intergroup *P* < 0.0001. *Post hoc* pairwise comparisons showed *P* = 0.0002 between the non-frail and pre-frail groups, and *P* < 0.0001 for the remaining pairwise comparisons (Figure 2). There were statistically significant intergroup differences in Aldrete score immediately upon PACU admission, PACU length of stay, incidence of prolonged stay, and time to achieve Aldrete score of 9 (all *P* < 0.001), while no statistically significant intergroup difference was observed in Aldrete score at PACU discharge (*P* = 0.062). The incidence of prolonged PACU stay increased from 13.9 to 51.0% with higher frailty grades. The Aldrete score immediately upon PACU admission gradually decreased from 7.6 ± 0.3 in the non-frail group to 6.5 ± 0.8 in the frail group, while the time required to achieve the Aldrete score of 9 recovery standard gradually increased. The incidence of nausea and vomiting, dizziness, drowsiness, postoperative hypotension, bradycardia, hypoxemia, and adverse events requiring pharmacological intervention in the PACU showed statistically significant intergroup differences (all *P* < 0.001), with the incidence of these adverse events continuing to rise with higher frailty grades. Stratified by the number of PACU adverse events, the proportion of patients with no adverse events decreased from 78.2% in the non-frail group to 44.0% in the frail group, while the proportion of patients with ≥2 adverse events increased from 3.0 to 20.0%, with an intergroup *P* < 0.001. The distribution of patient discharge disposition showed a statistically significant intergroup difference (*P* = 0.002), with the frail group having a higher proportion of prolonged observation and transfer to inpatient care compared with the other two groups (Table 3).

**Table 3:** Postoperative PACU recovery indicators and adverse events of patients by frailty status.

Characteristic	Total population (*n* = 500)	Non-frail group (*n* = 202)	Pre-frail group (*n* = 198)	Frail group (*n* = 100)	*P* value
Core PACU recovery indicators					
Aldrete score on PACU admission, mean ± SD	7.2 ± 0.6	7.6 ± 0.3	7.2 ± 0.5	6.5 ± 0.8	< 0.001
PACU length of stay (min), median (P25, P75)	28 (22, 35)	24 (20, 29)	28 (23, 34)	38 (31, 47)	< 0.001
Prolonged PACU length of stay, *n* (%)	125 (25.0)	28 (13.9)	46 (23.2)	51 (51.0)	< 0.001
Time to achieve Aldrete score of 9 (min), mean ± SD	26.8 ± 7.5	23.1 ± 5.2	26.5 ± 6.8	35.2 ± 8.7	< 0.001
Aldrete score at PACU discharge, mean ± SD	9.4 ± 0.4	9.5 ± 0.2	9.4 ± 0.5	9.3 ± 0.6	0.062
Adverse events in the PACU, *n* (%)					
Nausea and vomiting (WHO grade ≥1)					< 0.001
Yes	63 (12.6)	15 (7.4)	24 (12.1)	24 (24.0)	
No	437 (87.4)	187 (92.6)	174 (87.9)	76 (76.0)	
Dizziness					0.001
Yes	82 (16.4)	22 (10.9)	32 (16.2)	28 (28.0)	
No	418 (83.6)	180 (89.1)	166 (83.8)	72 (72.0)	
Drowsiness (arousable)					< 0.001
Yes	58 (11.6)	11 (5.4)	24 (12.1)	23 (23.0)	
No	442 (88.4)	191 (94.6)	174 (87.9)	77 (77.0)	
Hypotension (SBP<90 mmHg or > 30% decrease from baseline)					< 0.001
Yes	35 (7.0)	5 (2.5)	14 (7.1)	16 (16.0)	
No	465 (93.0)	197 (97.5)	184 (92.9)	84 (84.0)	
Bradycardia (HR<50 beats/min)					< 0.001
Yes	18 (3.6)	2 (1.0)	6 (3.0)	10 (10.0)	
No	482 (96.4)	200 (99.0)	192 (97.0)	90 (90.0)	
Hypoxemia (SpO_2_<90% lasting >10 s)					< 0.001
Yes	11 (2.2)	0 (0.0)	4 (2.0)	7 (7.0)	
No	489 (97.8)	202 (100.0)	194 (98.0)	93 (93.0)	
Adverse events requiring pharmacological intervention, *n* (%)					< 0.001
Yes	35 (7.0)	4 (2.0)	12 (6.1)	19 (19.0)	
No	465 (93.0)	198 (98.0)	186 (93.9)	81 (81.0)	
Total PACU adverse events, *n* (%)					< 0.001
0 events	326 (65.2)	158 (78.2)	124 (62.6)	44 (44.0)	
1 event	134 (26.8)	38 (18.8)	60 (30.3)	36 (36.0)	
≥2 events	40 (8.0)	6 (3.0)	14 (7.1)	20 (20.0)	
Discharge disposition, *n* (%)					0.002
Direct home	482 (96.4)	200 (99.0)	192 (97.0)	90 (90.0)	
Prolonged observation (> 1 h)	17 (3.4)	2 (1.0)	6 (3.0)	9 (9.0)	
Transfer to inpatient ward	1 (0.2)	0 (0.0)	0 (0.0)	1 (1.0)	

PACU, post-anesthesia care unit; SBP, systolic blood pressure; HR, heart rate; SpO_2_, pulse oxygen saturation; WHO, World Health Organization. Continuous variables are presented as mean ± SD or median (interquartile range), and categorical variables as *n* (%). For normally distributed continuous variables, one-way ANOVA was used for intergroup comparisons; for non-normally distributed continuous variables, Kruskal–Wallis *H* test was used; for categorical variables, chi-square test was used. Pairwise comparisons for categorical variables were performed using Bonferroni correction with a significance level of α' = 0.0167.

The overall incidence of total postoperative adverse events was statistically significantly different among the three groups (χ^2^ = 30.74, df = 2, *P* < 0.001). All pairwise comparisons after Bonferroni correction met the corrected test level. The incidence of total postoperative adverse events was 21.8% (95% CI: 16.2%−28.4%), 37.4% (95% CI: 30.5%−44.7%), and 56.0% (95% CI: 45.7%−65.9%) in the non-frail, pre-frail, and frail groups, respectively (Figure 1).

### 3.4 Univariate logistic regression analysis of factors associated with prolonged PACU length of stay

Univariate regression analysis was performed with prolonged PACU length of stay as the outcome variable. Age, BMI, ASA classification, frailty stratum, various chronic comorbidities, total number of comorbidities, per-kilogram doses of propofol and remifentanil, total procedure duration, intraoperative hypotension, intraoperative hypoxemia, and Aldrete score immediately upon PACU admission were correlated with the outcome (all *P* < 0.05). Increased age, higher ASA classification, higher frailty grade, increased number of chronic comorbidities, higher anesthetic drug dosages, longer endoscopic procedure duration, and intraoperative hypotension or hypoxemia increased the risk of prolonged PACU stay, while higher BMI and higher Aldrete score immediately upon PACU admission reduced this risk. Sex, type of endoscopic examination, intraoperative bradycardia, intraoperative movement, and intraoperative cough showed no statistically significant association with prolonged stay (all *P* > 0.05; Table 4).

**Table 4:** Univariate logistic regression analysis of factors associated with prolonged PACU length of stay.

Variable	Odds ratio (OR)	95% confidence interval (CI)		*P* value
		Lower limit	Upper limit	
Demographic characteristics				
Age (per 1-year increase)	1.123	1.078	1.169	< 0.001
Sex (female vs. male)	0.957	0.638	1.436	0.842
BMI (per 1 kg/m^2^ increase)	0.876	0.789	0.973	0.021
ASA classification				
Class I (reference)	1.000	–	–	–
Class II	2.137	1.176	3.885	0.012
Class III	5.258	2.557	10.806	< 0.001
Frailty status				
Non-frail (reference)	1.000	–	–	–
Pre-frail	1.867	1.108	3.146	0.019
Frail	6.418	3.607	11.419	< 0.001
Comorbidities				
Hypertension (yes vs. no)	2.346	1.537	3.578	< 0.001
Type 2 diabetes mellitus (yes vs. no)	2.119	1.358	3.302	0.001
Coronary heart disease (yes vs. no)	2.679	1.618	4.427	< 0.001
Chronic obstructive pulmonary disease (yes vs. no)	3.237	1.589	6.596	0.001
History of cerebrovascular disease (yes vs. no)	3.008	1.517	5.956	0.002
Number of comorbidities (per 1 increase)	1.678	1.409	1.997	< 0.001
Type of examination				
Isolated gastroscopy (reference)	1.000	–	–	–
Isolated colonoscopy	1.237	0.758	2.019	0.391
Combined gastroscopy and colonoscopy	1.557	0.928	2.609	0.092
Intraoperative characteristics				
Propofol dose per kg (per 0.1 mg/kg increase)	1.147	1.018	1.296	0.026
Remifentanil dose per kg (per 0.1 μg/kg increase)	1.208	1.029	1.417	0.020
Total procedure duration (per 5-min increase)	1.417	1.208	1.668	< 0.001
Intraoperative hypotension (yes vs. no)	2.867	1.728	4.759	< 0.001
Intraoperative bradycardia (yes vs. no)	1.987	0.916	4.309	0.082
Intraoperative movement reaction (yes vs. no)	1.765	0.923	3.376	0.086
Intraoperative cough (yes vs. no)	1.678	0.857	3.285	0.131
Intraoperative hypoxemia (yes vs. no)	3.557	1.508	8.387	0.004
Postoperative characteristic				
Aldrete score on PACU admission (per 1-point increase)	0.417	0.308	0.566	< 0.001

PACU, post-anesthesia care unit; BMI, body mass index; ASA, American Society of Anesthesiologists. Prolonged PACU length of stay was defined as PACU length of stay ≥35 min (the 75th percentile of the total population).

### 3.5 Multivariable logistic regression analysis of independent risk factors for prolonged PACU length of stay

**Figure 3:**
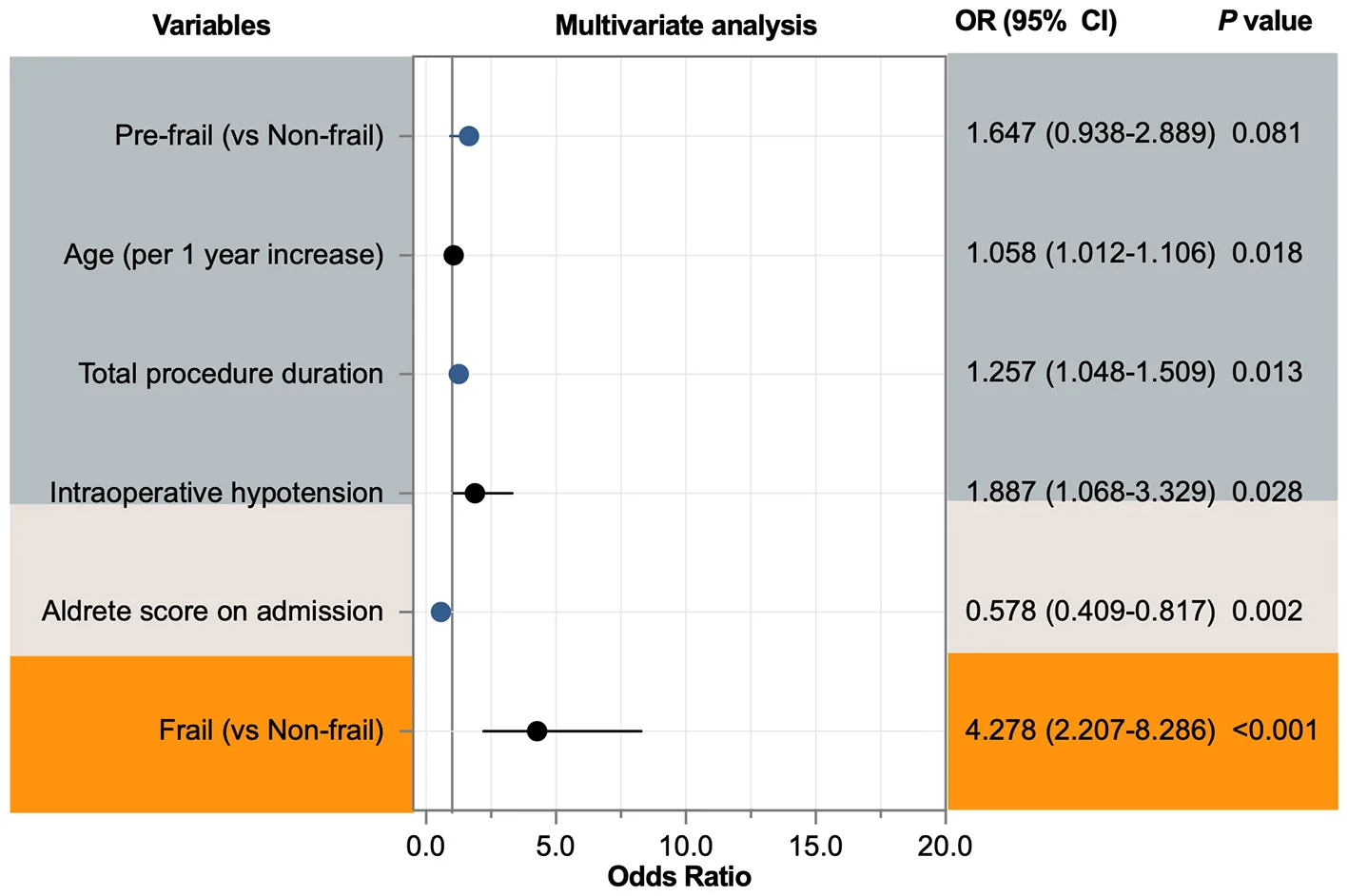
Forest plot of independent risk factors for prolonged PACU length of stay.

Variables with *P* < 0.05 in univariate analysis were included in the multivariable stepwise logistic regression model, and 5 indicators were finally identified as independent associated factors for prolonged PACU length of stay (Figure 3). Collinearity diagnosis confirmed no significant collinearity among variables in the final model, with all variance inflation factor values below 5 (detailed data shown in Supplementary Table S1). Age: for each 1-year increase in age, the risk of prolonged PACU stay increased (OR = 1.058, 95% CI: 1.012–1.106, *P* = 0.018). Frailty status: compared with the non-frail group, the pre-frail group showed no statistically significant difference (OR = 1.647, 95% CI: 0.938–2.889, *P* = 0.081), while frailty was an independent risk factor (OR = 4.278, 95% CI: 2.207–8.286, *P* < 0.001). Total procedure duration: for each 5-min prolongation, the risk of prolonged stay increased (OR = 1.257, 95% CI: 1.048–1.509, *P* = 0.013). Intraoperative hypotension: intraoperative hypotension increased the risk of prolonged stay (OR = 1.887, 95% CI: 1.068–3.329, *P* = 0.028). Aldrete score immediately upon PACU admission: for each 1-point increase in score, the risk of prolonged stay decreased (OR = 0.578, 95% CI: 0.409–0.817, *P* = 0.002; Table 5).

**Table 5:** Multivariable logistic regression analysis of independent risk factors for prolonged PACU length of stay.

Variable	Odds ratio (OR)	95% confidence interval (CI)		*P* value
		Lower limit	Upper limit	
Age (per 1-year increase)	1.058	1.012	1.106	0.018
Frailty status				
Non-frail (reference)	1.000	–	–	–
Pre-frail	1.647	0.938	2.889	0.081
Frail	4.278	2.207	8.286	< 0.001
Total procedure duration (per 5-min increase)	1.257	1.048	1.509	0.013
Intraoperative hypotension (yes vs. no)	1.887	1.068	3.329	0.028
Aldrete score on PACU admission (per 1-point increase)	0.578	0.409	0.817	0.002

PACU, post-anesthesia care unit. Variables with *P* < 0.05 in univariate analysis were included in the multivariable stepwise logistic regression model (forward method: inclusion criterion *P* < 0.05, exclusion criterion *P* > 0.10). The model was adjusted for age, frailty status, BMI, ASA classification, comorbidities, anesthetic drug dosages, total procedure duration, intraoperative adverse events, and Aldrete score on PACU admission.

### 3.6 Evaluation of the discriminative performance of the multivariable predictive model

**Figure 4:**
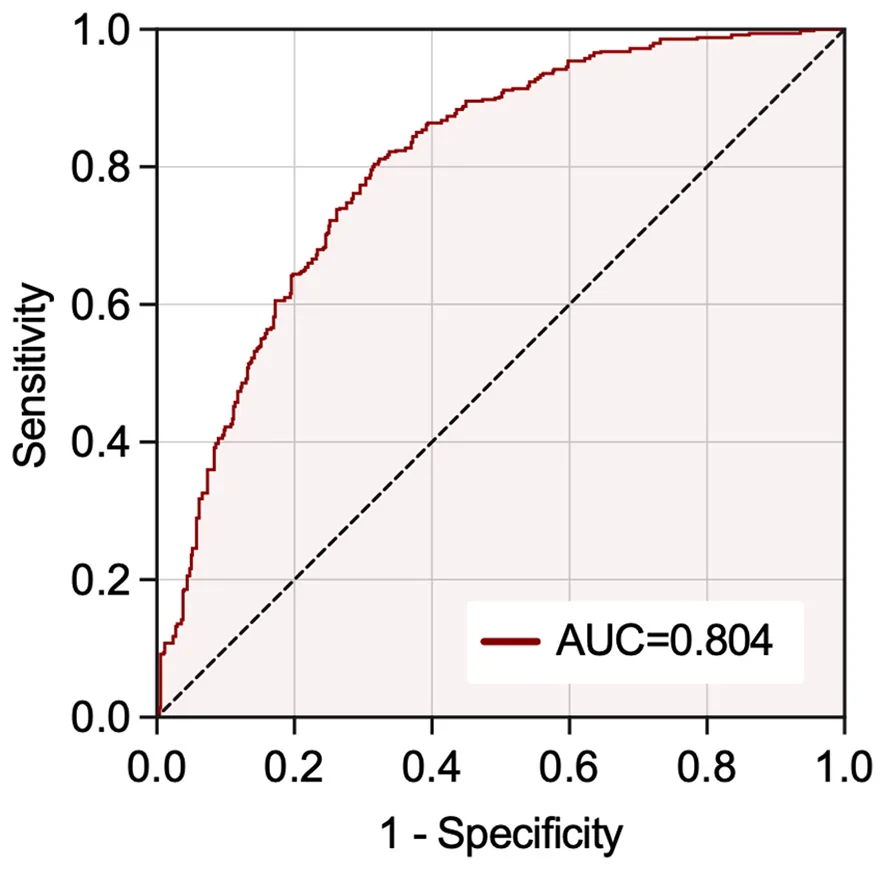
ROC curve of the multivariable predictive model for prolonged PACU length of stay.

A predictive model for prolonged PACU stay was constructed based on the variables included in the multivariable Logistic regression (age, frailty status, total procedure duration, intraoperative hypotension, and Aldrete score immediately upon PACU admission). The ROC curve was plotted with stay ≥35 min as the positive outcome. In the current derivation cohort, the model had an AUC of 0.804 (95% CI: 0.777–0.831); the optimal cutoff value was 0.36, corresponding to a model sensitivity of 82.4% and a specificity of 77.1% (Figure 4). Given the absence of internal and external validation, these performance metrics may have a degree of overestimation and require further confirmation in subsequent studies.

## 4 Discussion

This study systematically analyzed the association between frailty status and perioperative outcomes in 500 elderly outpatient patients undergoing painless gastrointestinal endoscopy. The results showed that frailty severity had a dose-dependent relationship with intraoperative anesthetic drug requirements, procedure duration, incidence of intraoperative and postoperative adverse events, and PACU length of stay. Multivariable regression analysis confirmed that frailty was an independent risk factor for prolonged PACU length of stay, and the multivariable predictive model incorporating frailty status constructed on this basis had favorable discriminative performance for prolonged PACU stay.

Previous studies on frailty and perioperative outcomes in digestive endoscopy have mostly analyzed frailty as a dichotomous variable, only comparing differences between frail and non-frail patients and neglecting pre-frailty as an important transitional stage (25). This study used the internationally recognized Fried Frailty Phenotype criteria to divide patients into non-frail, pre-frail, and frail groups (26). The results showed that with increasing frailty severity, patients were older, had more comorbidities, and higher ASA classifications, consistent with the pathophysiological characteristics of frailty as a progressive decline in multisystem physiological reserve. Regarding anesthetic drug dosages, the per-kilogram doses of propofol and remifentanil gradually decreased with higher frailty grades. Since all patients received the same protocolized titration to target sedation depth rather than pre-set dose reduction, this difference reflects the increased central nervous system sensitivity to anesthetics and impaired hepatic metabolic function in frail elderly patients, which is consistent with the pharmacokinetic and pharmacodynamic characteristics of aging and frailty. This study also found that the incidence of intraoperative and postoperative adverse events and PACU length of stay were significantly higher in pre-frail patients compared with non-frail patients. Although pre-frailty did not reach statistical significance after multivariable adjustment (OR = 1.647, *P* = 0.081), it still indicated a potential risk of delayed postoperative recovery. This result is consistent with the conclusion of Zhang et al. (27), suggesting that pre-frailty, as an early stage of decreased physiological reserve, also increases the risk of perioperative adverse events. Clinical practice should not only focus on patients with definite frailty but also attach importance to perioperative management of the pre-frail population.

The multivariable analysis results of this study showed that in addition to frailty, age, total procedure duration, intraoperative hypotension, and Aldrete score immediately upon PACU admission were also independent risk factors for prolonged PACU length of stay. For each 1-year increase in age, the risk of prolonged PACU stay increased by 5.8%, which is related to multisystem physiological degenerative changes and impaired drug metabolism and clearance in elderly patients. For each 5-min prolongation of total procedure duration, the risk of prolonged stay increased by 25.7%. Prolonged procedure duration not only increases the total exposure to anesthetic drugs but also leads to enhanced stress response and higher incidence of intraoperative adverse events, thereby affecting postoperative recovery. Intraoperative hypotension increased the risk of prolonged PACU stay by 88.7%. Hypotension-induced insufficient tissue and organ perfusion, especially reduced cerebral perfusion, prolongs postoperative recovery time and increases the risk of postoperative adverse events such as dizziness and drowsiness (28, 29). For each 1-point increase in Aldrete score immediately upon PACU admission, the risk of prolonged stay decreased by 42.2%. This score directly reflects the patient's immediate postoperative recovery status and is a sensitive indicator for predicting PACU length of stay. Notably, BMI, ASA classification, and various chronic comorbidities that were statistically significant in univariate analysis were no longer independent risk factors after incorporating frailty status. This finding is partially attributed to the collinearity between frailty and these traditional risk factors; frailty, as a comprehensive indicator for assessing multisystem physiological reserve, integrates information from age, comorbidity burden, and functional status, thus demonstrating stronger independent predictive value for postoperative recovery in elderly patients compared with single ASA classification or comorbidity assessment (30, 31).

Currently, there are few predictive models for prolonged PACU stay in elderly patients undergoing painless gastrointestinal endoscopy, and existing models mostly do not incorporate frailty status as a key factor (32). The multivariable predictive model constructed in this study, which includes age, frailty status, total procedure duration, intraoperative hypotension, and Aldrete score immediately upon PACU admission, had an AUC of 0.804. The optimal cutoff value of 0.36 corresponded to a sensitivity of 82.4% and a specificity of 77.1%. All indicators of this model are routinely available clinical parameters without the need for additional complex examinations, making it suitable for popularization and application in endoscopy centers at all levels. Given that prolonged PACU stay is linked to both reduced operational efficiency of endoscopy units and elevated risk of postoperative adverse events, preoperative application of this model can quickly identify high-risk patients for prolonged PACU stay, helping to optimize medical resource allocation: for low-risk patients, a fast-track resuscitation process can be adopted to shorten PACU length of stay; for high-risk patients, PACU beds can be reserved in advance, and intraoperative and postoperative monitoring can be strengthened to reduce the risk of adverse events. This is consistent with the research objective of this study to propose a personalized postoperative management strategy based on frailty assessment.

Based on the above research results, this study provides a basis for the perioperative management of elderly patients undergoing painless gastrointestinal endoscopy. It is recommended that Fried Frailty Phenotype assessment be incorporated into the routine preoperative evaluation process for elderly patients undergoing painless gastrointestinal endoscopy, and individualized anesthesia regimens be formulated according to frailty status: for frail patients, anesthetic drug dosages should be appropriately reduced, and drugs with less impact on respiratory and circulatory functions should be preferentially selected. For frail patients, endoscopic procedure duration should be minimized as much as possible, and intraoperative blood pressure monitoring and management should be strengthened to avoid prolonged hypotension. For frail patients and those assessed as high-risk by the predictive model, postoperative PACU monitoring time should be prolonged, and recovery period nursing should be enhanced to reduce the risk of postoperative adverse events.

This study has certain limitations. First, this was a single-center retrospective study, and all subjects were from Cangzhou area, Hebei Province, which may have selection bias, and the generalizability of the research results needs further verification. Although data were recorded using pre-designed CRF forms during the study period, information bias inherent to retrospective studies cannot be completely avoided. Second, this study only evaluated short-term perioperative outcomes and did not analyze the impact of frailty on 30-day postoperative and long-term quality of life. Third, potential factors that may affect postoperative recovery, such as cognitive function, nutritional status, and social support, were not included. Fourth, only the apparent discriminative performance of the predictive model was evaluated in the single-center derivation cohort, and neither internal validation (e.g., Bootstrap resampling) nor external validation with an independent cohort was performed. The model may have a certain degree of overfitting, and its generalizability and stability need further confirmation in multicenter studies with larger sample sizes. Fifth, the cutoff value for prolonged PACU length of stay was derived from the 75th percentile of this single-center cohort, and the generalizability of the 35-min threshold to other medical centers with different workflow configurations requires further verification.

## 5 Conclusion

Frailty is an independent risk factor for prolonged PACU length of stay in elderly patients undergoing painless gastrointestinal endoscopy, and pre-frail patients also have a potential risk of delayed postoperative recovery. The multivariable predictive model constructed based on frailty status has favorable discriminative performance for prolonged PACU stay and is helpful for clinical preoperative risk stratification and individualized management. Future multicenter prospective studies are needed to further validate the results of this study and explore the effectiveness of frailty assessment-based interventions in improving perioperative outcomes in elderly patients.

## Data Availability

The original contributions presented in the study are included in the article/Supplementary material, further inquiries can be directed to the corresponding author.
