# Supplementary material for: Correlation between frailty status and prolonged length of stay in the post-anesthesia care unit and development of a predictive model in elderly patients undergoing painless gastrointestinal endoscopy
Source: Front Med (Lausanne). 2026 Aug 10;13:1905761. doi: 10.3389/fmed.2026.1905761 (PMC13500554; doi:10.3389/fmed.2026.1905761)
Supplement: Supplementary file 1 [file Data_Sheet_1.zip › Supplementary Materials/Supplementary Table 2.docx]

**Supplementary Table 2. Missing rate of core analysis variables before imputation (n=500).**

| **Variable Category** | **Variable Name** | **Number of Missing Cases** | **Missing Rate (%)** |
| --- | --- | --- | --- |
| Baseline characteristics | Age | 0 | 0.0 |
|  | Sex | 0 | 0.0 |
|  | Body mass index (BMI) | 2 | 0.4 |
|  | ASA classification | 1 | 0.2 |
|  | Smoking history | 0 | 0.0 |
|  | Drinking history | 0 | 0.0 |
|  | Hypertension | 0 | 0.0 |
|  | Type 2 diabetes mellitus | 0 | 0.0 |
|  | Coronary heart disease | 1 | 0.2 |
|  | Chronic obstructive pulmonary disease | 1 | 0.2 |
|  | History of cerebrovascular disease | 2 | 0.4 |
| Intraoperative parameters | Total propofol dose | 1 | 0.2 |
|  | Total remifentanil dose | 1 | 0.2 |
|  | Total procedure duration | 2 | 0.4 |
|  | Intraoperative hypotension | 0 | 0.0 |
|  | Intraoperative hypoxemia | 0 | 0.0 |
| PACU outcomes | Aldrete score on PACU admission | 1 | 0.2 |
|  | PACU length of stay | 0 | 0.0 |
|  | Prolonged PACU length of stay | 0 | 0.0 |
