# Supplementary material for: Correlation between frailty status and prolonged length of stay in the post-anesthesia care unit and development of a predictive model in elderly patients undergoing painless gastrointestinal endoscopy
Source: Front Med (Lausanne). 2026 Aug 10;13:1905761. doi: 10.3389/fmed.2026.1905761 (PMC13500554; doi:10.3389/fmed.2026.1905761)
Supplement: Supplementary file 1 [file Data_Sheet_1.zip › Supplementary Materials/Supplementary Table 1.docx]

**Supplementary Table 1. Variance inflation factor (VIF) of variables in the final multivariable logistic regression model.**

| **Variable** | **VIF** |
| --- | --- |
| Age (years) | 1.84 |
| Frailty status | 1.72 |
| Total procedure duration | 1.21 |
| Intraoperative hypotension | 1.35 |
| Aldrete score on PACU admission | 1.46 |
